# Remote ischemic preconditioning and its role in the prevention of new onset atrial fibrillation post‐cardiac surgery. A meta‐analysis of randomized control trials

**DOI:** 10.1002/joa3.12252

**Published:** 2019-10-31

**Authors:** Ashish Kumar, Harpreet Singh, Mariam Shariff

**Affiliations:** ^1^ Department of Critical Care Medicine St John’s Medical College Hospital Bangalore India; ^2^ Internist Michigan Primary Care Partners Rapids MI USA

**Keywords:** meta‐analysis, new onset atrial fibrillation, remote ischemic preconditioning

## Abstract

**Background:**

The denouement of remote ischemic preconditioning on new onset atrial fibrillation (NOAF) post‐cardiac surgery is not well‐established. An updated meta‐analysis of randomized control trials was performed by comparing remote ischemic preconditioning with controls and the outcome of interest was NOAF.

**Methods:**

The systemic review was performed in accordance with the PRISMA (Preferred reporting items for systemic review) and AHA (American Heart Association) guidelines**.** PubMed database was searched to include relevant randomized control trials from inception to July 2019. We used Mantel‐Haenzsel method with random error model to calculate risk ratio (RR) with 95% confidence interval (CI). Heterogeneity was assessed using the *I* ^2^ test> 50% or *χ* ^2^ *P* < .05. Publication bias was visually assessed using a funnel plot.

**Results:**

Twelve randomized control trials were included in the final analysis. Remote ischemic preconditioning did not alter the risk of NOAF post‐cardiac surgery [RR: 0.95, CI: 0.83‐1.09, *P* = .48, *I* ^2^ = 37%, *χ* ^2^ *P* = .09].

**Conclusion:**

In conclusion, the present meta‐analysis failed to provide any evidence for the beneficial effect of remote ischemic preconditioning in the prevention of NOAF.

## Graphical abstract

The evidence from the present meta‐analysis does not support the beneficial effect of remote ischemic preconditioning in preventing new onset atrial fibrillation post‐cardiac surgery.

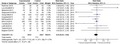

## 1 INTRODUCTION

The incidence of atrial fibrillation post‐cardiac surgery is estimated to be around 30%‐40%.^1^ Atrial fibrillation post‐cardiac surgery, being associated with increased morbidity and mortality, is also associated with increased utilization of medical resources and surged health‐care cost. Consequently, there has been a quest toward the need for an ideal and noninvasive method for the prevention of atrial fibrillation post‐cardiac surgery. Drugs like beta blockers have been studied and scrutinized for prevention of atrial fibrillation post‐cardiac surgery with commendatory results.^2^ Remote ischemic preconditioning technique has recently gained popularity as a method to prevent ischemic reperfusion injury during cardiac surgery. The technique consists of inducing brief episodes of ischemia followed by reperfusion in a remote vascular territory or an organ. The technique has also been studied to prevent acute kidney injury following cardiac surgery.^3^ However, the evidence regarding the effect of remote ischemic preconditioning on the risk of atrial fibrillation post‐cardiac surgery is controversial. Several meta‐analyses in the field have failed to analyze the effect of remote ischemic preconditioning on the risk of new onset atrial fibrillation (NOAF) post‐cardiac surgery. Therefore, we performed an updated meta‐analysis of randomized control trials by comparing remote ischemic preconditioning with controls and the outcome of interest was NOAF, for pooled estimation in meta‐analysis.

## 2 METHODS

**Figure 1:**
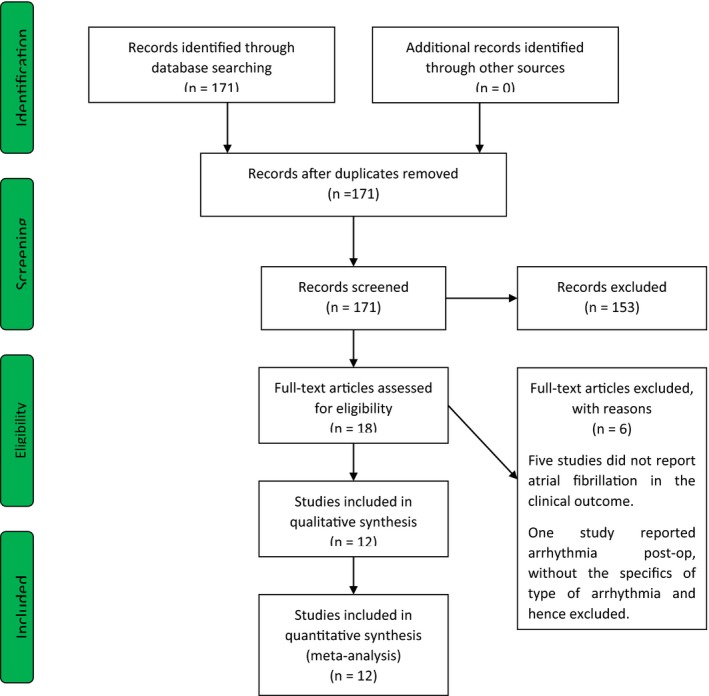
PRISMA flow chart

The systemic review was performed in accordance with the PRISMA (Preferred reporting items for systemic review) and AHA (American heart association) guidelines.^4^, ^5^ We performed a systematic search through PubMed database to identify relevant randomized control trials from inception to July 2019. The following terms were used for systematic search in the PubMed database—"remote ischemic pre‐condition*, "remote ischemic precondition*", "cardiac surgery", "bypass‐surgery", "bypass", "surgical aortic valve replacement", SAVR. The search strategy is further elaborated in the supplementary file. The inclusion criteria for studies were: randomized control trials studying the effect of remote ischemic preconditioning juxtaposed to controls in subjects undergoing cardiac surgery and reporting the incidence of NOAF. Articles were not excluded based on sample size. Only manuscripts published in English were considered for final analysis. The database search was augmented with manual search of bibliographies of included articles, to include relevant articles not identified by database search. The PRISMA flow chart for inclusion of studies is depicted in Figure 1.

Two authors AK and MS independently screened the abstracts to include relevant articles and performed data extraction. Any disparity was resolved by mutual consensus. Data extraction was performed in accordance with a standardized predefined data extraction form. The following data were extracted from each study: author's name, year of study, study design, number randomized, mean age, percentage male, primary outcomes of interest, number of individuals with NOAF in the intervention and control group.

We used Mantel‐Haenzsel method with random error model to calculate risk ratio (RR) with 95% confidence interval (CI). Heterogeneity was assessed using the *I* ^2^ test > 50% or *χ* ^2^ *P *< .05. Publication bias was visually assessed using funnel plot. The analysis was carried out using RevMan Version 5.3. (Copenhagen: The Nordic Cochrane Centre, The Cochrane Collaboration, 2014).

## 3 RESULTS

The systematic search unveiled a total of 171 eligible articles. Twelve randomized control trials were included in the final analysis.^3^, ^6^, ^7^, ^8^, ^9^, ^10^, ^11^, ^12^, ^13^, ^14^, ^15^, ^16^ This sums up to a total of 2652 procedures in the remote ischemic preconditioning group and 2667 procedure in the control group. There were three prominent studies of the 12 included studies,^10^, ^14^, ^16^ which together constituted more than 75% of the patients in the final analysis. Baseline characteristics of included studies are shown in Table 1. The exact technique of remote ischemic preconditioning used in each included trial has also been outlined in Table 1. Of the 12 randomized control trials included, only eight trials randomized patients undergoing coronary artery bypass surgery. The PRISMA checklist is provided in the supplementary file (Table S1).

**Table 1:** Baseline characteristics of included studies

Study	Year	Study design	Number randomized (intervention/control)	Participant selection	Method of remote ischemic preconditioning	Mean age (intervention/control) in years	Percentage male (intervention/control) %	Primary outcome of interest in the study
Bagheri et al	2018	RCT	87/90	Patients undergoing CABG	The RIPC group received three sequential sphygmomanometer cuff inflations on their right upper arms. There was a gap of 5 minutes between each inflations	63/64	60/56	Acute kidney Injury
Tuter et al	2018	RCT	40/40	Patients undergoing CABG	Three cycles of 10 minutes of ischemia were applied to the right lower limb at the level of the upper third of the thigh by inflation of a blood pressure cuff to 200 mm Hg, followed by 10 minutes reperfusion while the cuff was deflated	64/64	82/77	Primary endpoint was serum concentration of troponin I and lactate 2 and 24 hours after surgery.
Lotfi et al	2016	RCT	51/51	Patients undergoing CABG	Treatment group received three sequential sphygmomanometer cuff inflations on their right upper arm after induction of anesthesia. Each inflation and deflation lasted for 5 minutes	69/69	76/63	New onset atrial fibrillation
Candilio et al	2015	RCT	89/89	Patients undergoing CABG or valve surgery.	5 minutes cycles of simultaneous upper arm and thigh cuff inflation/deflation, two cycles, Cuff pressure raised to 200 mm Hg	65/66	81/75	Perioperative myocardial injury
ERICCA	2015	RCT	779/794	Patients undergoing CABG with EuroSCORE 5 or higher.	A standard blood‐pressure cuff was placed on the upper arm, inflated to 200 mm Hg, and left inflated for 5 minutes, followed by 5 minutes deflation, four cycles	76/76	70/73	Combined primary end point of death from cardiovascular causes, nonfatal myocardial infarction, coronary revascularization, or stroke
RIPHeart	2015	RCT	690/690	Patients undergoing elective cardiovascular surgery	5‐minute blood‐pressure cuff inflation to ≥200 mm Hg, but at least 15 mm Hg higher than the patient's actual systolic arterial pressure, followed by 5‐minute cuff deflation, four cycles	66/66	75/73	Composite of death, myocardial infarction, stroke, or acute renal failure
Krogstad et al	2015	RCT	45/47	Patients undergoing CABG	The RIPC stimulus comprised three 5‐min cycles of upper arm ischemia, induced by inflating a blood pressure cuff to 200 mm Hg, with an intervening 5 minutes reperfusion, three cycles	64/64	93/91	New onset atrial fibrillation
Hong et al	2014	RCT	644/636	Patients undergoing elective cardiac surgery	The cuff was inflated to 200 mm Hg for 5 minutes and deflated for 5 minutes. This inflation‐deflation cycle was repeated four times. This inflation‐deflation protocol was applied twice immediately after induction of anaesthesia	61/61	61/61	Major adverse cardiac events
Slagsvold et al	2014	RCT	30/30	Patients undergoing CABG	RIPC was performed preoperatively by inflating a blood pressure cuff on the upper arm to 200 mm Hg for 3 × 5 minutes, with 5 minutes reperfusion intervals	64/68	90/77	Mitochondrial respiration
Meybohm et al	2013	RCT	90/90	Patients undergoing cardiac surgery	5‐minute blood pressure cuff inflation to 200 mm Hg, a cuff‐pressure at least 15 mm Hg higher than the systolic arterial pressure measured via the arterial line, and 5‐min cuff deflation, four cycles	70/68	77/85	Postoperative neurocognitive dysfunction
Lucchinetti et al	2012	RCT	27/28	Patients undergoing CABG	Four 5‐minute cycles of 300 mm Hg cuff inflation/deflation of the leg before aortic cross‐clamping	59/62	96/86	High‐sensitivity cardiac troponin T
Rahman et al	2010	RCT	80/82	Patients undergoing CABG	Upper limb (, 5‐minute cycles of 200 mm Hg cuff inflation/deflation, three cycles	63/65	89/88	Troponin T (cTnT)

Abbreviations: CABG, coronary artery bypass graft; RCT, randomized control trial; RIPC, remote ischemic preconditioning.

**Figure 2:**
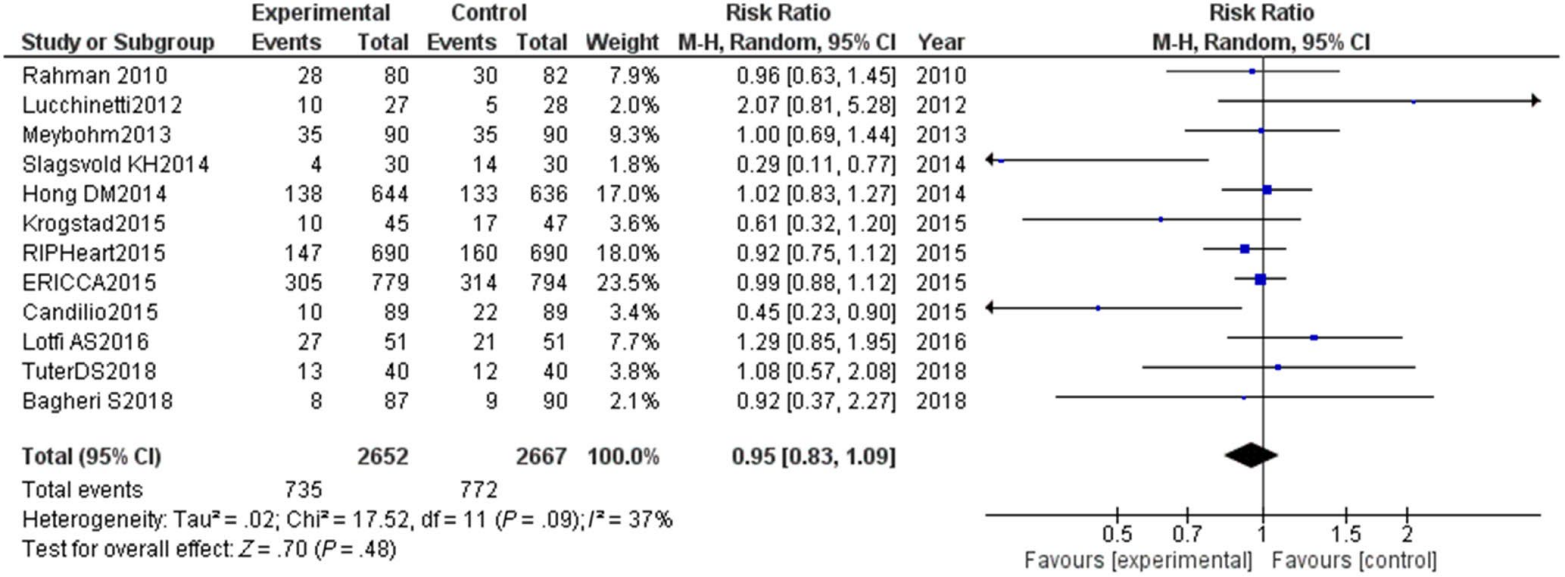
Forest plot for new onset atrial fibrillation(NOAF) post‐cardiac surgery. The experimental group depicted in the forest plot was the group who underwent remote ischemic preconditioning. M‐H; Mantel‐Haenzsel method,CI; confidence interval

Remote ischemic preconditioning did not alter the risk of NOAF post‐cardiac surgery [RR: 0.95, CI: 0.83‐1.09, *P* = .48, *I* ^2^ = 37%, *χ* ^2^ *P* = .09] (Figure 2). There was no heterogeneity associated with the pooled estimate as evident from the *I* ^2^ and *χ* ^2^ *P*‐value. Visual inspection of the funnel plot did not depict publication bias (Figure S1).

## 4 DISCUSSION

An updated meta‐analysis comparing remote ischemic preconditioning with controls using the data from 12 randomized control trials with 2652 procedures in the intervention arm and 2667 procedures in the control arm was performed. The main result of this meta‐analysis concluded that remote ischemic preconditioning prior to cardiac surgery did not curtail the risk of NOAF. To our knowledge, this was the first meta‐analysis researching the effect of remote ischemic preconditioning on NOAF, prior to cardiac surgery.

A study by Krogstad et al had similar conclusion as our meta‐analysis which found no difference in the incidence of NOAF among the remote ischemic precondition group as compared to the control group undergoing cardiac surgery.^15^ Besides, the three large trials, studying the effect of remote ischemic preconditioning on clinical outcomes in patients undergoing cardiac surgery, namely the RIPHeart trail, the ERICCA trial, and the study by Hong et al, found no beneficial effect of remote ischemic preconditioning on the incidence of NOAF post‐surgery.^10^, ^14^, ^16^

The results of this meta‐analysis are however incongruous with the results of a randomized control trial which concluded that remote ischemic preconditioning reduced the inducibility and sustainability of nonvalvular atrial fibrillation. Additionally, the study also concluded that these changes were possibly mediated by alteration in the electrophysiological properties of the atria.^17^ In a study by Candilio et al, remote ischemic precondition significantly reduced the incidence of NOAF among subjects undergoing cardiac surgery.^6^ Furthermore, Candilio et al also concluded that remote ischemic preconditioning reduced perioperative myocardial injury in patients undergoing cardiac surgery, which could have contributed to the reduced incidence of NOAF. Supporting the previous study was a study by Slagsvold et al, which concluded that remote ischemic preconditioning minimized the incidence of NOAF among subjects undergoing coronary artery bypass graft. The study further concluded that this reduction in incidence of NOAF can be accredited to preserved mitochondrial function by remote ischemic preconditioning and its influence on myocardial MicroRNA (miR) expression of the atrial myocardium among subjects undergoing coronary artery bypass surgery.^9^ The study adumbrated regarding the prevention of miR upregulation by remote ischemic preconditioning prior to cardiac surgery. Increased miR expression has been associated with greater extent of myocardial injury following ischemia reperfusion injury.^18^ Disarray in the functioning of mitochondria increases the risk of disturbance in the homeostasis of electrolytes and cardiac arrhythmia.^9^ Further in another study by Slagsvold et al, the authors demonstrated the cardioprotective effect of remote ischemic preconditioning by preserving the mitochondrial activity and activation of the protein kinase akt in left ventricle of the patients undergoing coronary artery bypass surgery. It has been postulated that activation of protein kinase akt plays a beneficial role in the survival of myocardial cells during cardiac surgery.^19^ The possible role of remote ischemic preconditioning in altering the ionic distribution along the cellular component of the atria has also been postulated.^17^

There are several limitations in our analysis. First, we have not attributed in our analysis the biases that could be associated with each randomized control trial. Second, we have concentrated only on NOAF and not analyzed the effect of remote ischemic precondition on other types of arrhythmias. Third, different anesthetic agents used during cardiac surgery are known to have varied effect on the risk of NOAF post‐cardiac surgery and has not been attributed in our present analysis. Fourth, this is a study level meta‐analysis and future patient level meta‐analysis would provide better evidence. Finally, the method used for inducing remote ischemic preconditioning varied slightly in each trial and has not been attributed in the present analysis.

In conclusion, the present meta‐analysis of randomized control trials did not delineate any beneficial effect of remote ischemic preconditioning on the risk of NOAF.

## CONFLICT OF INTEREST

Authors declare no conflict of interests for this article.

## References

[1] Mathew JP , Fontes ML , Tudor IC , Ramsay J , Duke P , Mazer CD , et al. A multicenter risk index for atrial fibrillation after cardiac surgery. JAMA. 2004;291(14):1720–9. 10.1001/jama.291.14.1720 15082699

[2] Mostafa A , El‐Haddad MA , Shenoy M , Tuliani T . Atrial fibrillation post cardiac bypass surgery. Avicenna J Med. 2012;2(3):65–70. https://www.ncbi.nlm.nih.gov/pubmed/23826549 23826549 10.4103/2231-0770.102280 PMC3697424

[3] Bagheri S , Shahbazi S , Shafa M , Borhani‐Haghighi A , Kiani M , Sagheb MM . The effect of remote ischemic preconditioning on the incidence of acute kidney injury in patients undergoing coronary artery bypass graft surgery: a randomized controlled trial. Iran J Med Sci. 2018;43(6):587–95. https://www.ncbi.nlm.nih.gov/pubmed/30510335 30510335 PMC6230941

[4] Moher D , Liberati A , Tetzlaff J , Altman DG ; PRISMA Group . Preferred reporting items for systematic reviews and meta‐analyses: the PRISMA statement. PLoS Medicine. 2009;6(7):e1000097 https://www.ncbi.nlm.nih.gov/pubmed/19621072 19621072 10.1371/journal.pmed.1000097 PMC2707599

[5] Rao G , Lopez‐Jimenez F , Boyd J , D'Amico F , Durant NH , Hlatky MA , et al. Methodological standards for meta‐analyses and qualitative systematic reviews of cardiac prevention and treatment studies: a scientific statement from the American Heart Association. Circulation. 2017;136(10):e172–e194. 10.1161/CIR.0000000000000523 28784624

[6] Candilio L , Malik A , Ariti C , Barnard M , Di Salvo C , Lawrence D , et al. Effect of remote ischaemic preconditioning on clinical outcomes in patients undergoing cardiac bypass surgery: a randomised controlled clinical trial. Heart. 2015;101(3):185–92. https://heart.bmj.com/content/101/3/185.abstract 25252696 10.1136/heartjnl-2014-306178

[7] Rahman IA , Mascaro JG , Steeds RP , Frenneaux MP , Nightingale P , Gosling P , et al. Remote ischemic preconditioning in human coronary artery bypass surgery. Circulation. 2010;122(11_suppl_1):S53–S59. 10.1161/CIRCULATIONAHA.109.926667 20837926

[8] Lucchinetti E , Bestmann L , Feng J , Freidank H , Clanachan AS , Finegan BA , et al. Remote ischemic preconditioning applied during isoflurane inhalation provides no benefit to the myocardium of patients undergoing on‐pump coronary artery bypass graft surgery. Anesthesiology. 2012;116(2):296–310. 10.1097/ALN.0b013e318242349a 22222469

[9] Hordnes SK , Øivind R , Morten H , Ulrik W , Alexander W . Remote ischemic preconditioning preserves mitochondrial function and influences myocardial microRNA expression in atrial myocardium during coronary bypass surgery. Circ Res. 2014;114(5):851–9. 10.1161/CIRCRESAHA.114.302751 24371264

[10] Hong DM , Lee E‐H , Kim HJ , Min JJ , Chin J‐H , Choi D‐K , et al. Does remote ischaemic preconditioning with postconditioning improve clinical outcomes of patients undergoing cardiac surgery? Remote ischaemic preconditioning with postconditioning outcome trial. Eur Heart J. 2014;35(3):176–83. 10.1093/eurheartj/eht346 24014392

[11] Tuter DS , Kopylov PY , Syrkin AL , Glazachev OS , Komarov RN , Katkov AI , et al. Intermittent systemic hypoxic–hyperoxic training for myocardial protection in patients undergoing coronary artery bypass surgery: first results from a single‐centre, randomised controlled trial. Open Heart. 2018;5(2):e000891 https://openheart.bmj.com/content/5/2/e000891 30487981 10.1136/openhrt-2018-000891 PMC6241980

[12] Meybohm P , Renner J , Broch O , Caliebe D , Albrecht M , Cremer J , et al. Postoperative neurocognitive dysfunction in patients undergoing cardiac surgery after remote ischemic preconditioning: a double‐blind randomized controlled pilot study. PLoS ONE. 2013;8(5):e64743 https://www.ncbi.nlm.nih.gov/pubmed/23741380 23741380 10.1371/journal.pone.0064743 PMC3669352

[13] Lotfi AS , Eftekhari H , Atreya AR , Kashikar A , Sivalingam SK , Giannoni M , et al. Randomized controlled trial of remote ischemic preconditioning and atrial fibrillation in patients undergoing cardiac surgery. World J Cardiol. 2016;8(10):615–22. https://www.ncbi.nlm.nih.gov/pubmed/27847563 27847563 10.4330/wjc.v8.i10.615 PMC5088368

[14] Meybohm P , Bein B , Brosteanu O , Cremer J , Gruenewald M , Stoppe C , et al. A multicenter trial of remote ischemic preconditioning for heart surgery. N Engl J Med. 2015;373(15):1397–407. 10.1056/NEJMoa1413579 26436208

[15] Krogstad L , Slagsvold KH , Wahba A . Remote ischemic preconditioning and incidence of postoperative atrial fibrillation. Scand Cardiovasc J. 2015;49(3):117–22. 10.3109/14017431.2015.1010565 25613907

[16] Hausenloy DJ , Candilio L , Evans R , Ariti C , Jenkins DP , Kolvekar S , et al. Remote ischemic preconditioning and outcomes of cardiac surgery. N Engl J Med. 2015;373(15):1408–17. 10.1056/NEJMoa1413534 26436207

[17] Kosiuk J , Langenhan K , Stegmann C , Uhe T , Dagres N , Dinov B , et al. Effect of remote ischemic preconditioning on electrophysiological parameters in nonvalvular paroxysmal atrial fibrillation: The RIPPAF Randomized Clinical Trial. Heart Rhythm. 2019; In press. 10.1016/j.hrthm.2019.07.026 31356986

[18] Cheng Y , Tan N , Yang J , Liu X , Cao X , He P , et al. A translational study of circulating cell‐free microRNA‐1 in acute myocardial infarction. Clin Sci. 2010;119(2):87–95. https://www.clinsci.org/content/119/2/87 20218970 10.1042/CS20090645 PMC3593815

[19] Slagsvold KH , Moreira J , Rognmo Ø , Høydal M , Bye A , Wisløff U , et al. Remote ischemic preconditioning preserves mitochondrial function and activates pro‐survival protein kinase Akt in the left ventricle during cardiac surgery: a randomized trial. Int J Cardiol. 2014;177(2):409–17. 10.1016/j.ijcard.2014.09.206 25456576

