# Supplementary material for: Remote ischemic preconditioning and its role in the prevention of new onset atrial fibrillation post‐cardiac surgery. A meta‐analysis of randomized control trials
Source: J Arrhythm. 2019 Oct 31;35(6):789–94. doi: 10.1002/joa3.12252 (PMC6898524; doi:10.1002/joa3.12252)

**Search strategy used in PubMed**

((("remote ischemic pre-condition*" OR "remote ischemic precondition*"))) AND (("cardiac surgery" OR "bypass surgery" OR "bypass" OR "surgical aortic valve replacement" OR SAVR))

Search performed till July31,2019.

**Figure S1**- Funnel plot for new onset atrial fibrillation (NOAF) post cardiac surgery.


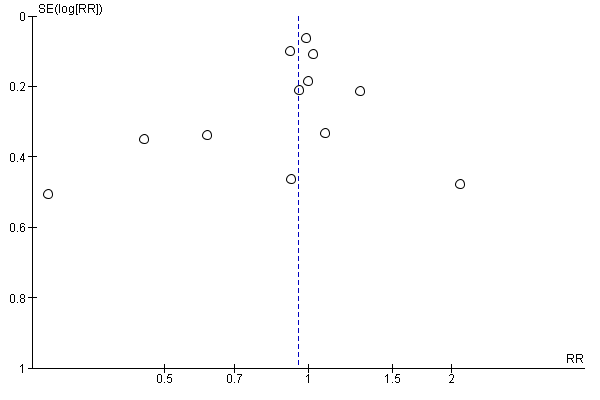

Supplement: Supplementary file 1 [file JOA3-35-789-s001.docx]
